# Indents

**Published:** 1907-09-15

**Authors:** 


### INDENTS.

☞ **Brief Articles of Technical or Scientific Value will receive notice and comment. Contributions invited.**

**Nose Blowing.** Some people blow the nose entirely too hard. There are cases on record in which damage has been done to the ears in this way, and other accessory cavities may be injured. Rhinologists are very careful about this. One man in Philadelphia advises his patients not to blow the nose at all. I try to teach mine to do as the Irishman does, hold one side and blow the other gently in the seclusion of their own room and over a bowl, of course. In this way there can be no danger, unless perchance one side should be occluded by some thickening of the tissue. Many people blow the nose unnecessarily and when there is nothing to remove, and in this way they only irritate the sensitive mucous membrane.—Dr. Makum, in *Dental Brief.*

**Diseased Molar Socket.** In a case in which there was a profuse discharge of pus from the socket of a molar serving as abutment for a bridge, without removing the bridge the socket was restored to health, and the abutment made firm by the following treatment: Using the compressed air atomizer with a Parke-Davis hypodermic needle as a nozzle, the socket was washed once a day with the following preparation:

| | |
|---|---|
| ℞—Campho-phenique | ʒj |
| Dioxogen | ℥j |
| Glyco-thymolin | ℥iij |
| Water, q. s. ad | ℥vj. |

Twelve treatments were required.—P. Neff Myers, *Dental Cosmos.*

**Sulpho-Carbolate of Zinc for Pyorrhea.**

I desire to present the merits of sulpho-carbolate of zinc as a medicament of great efficiency. It is obtained by heating a mixture of carbolic acid and sulphuric acid and saturating the product with oxide of zinc, then evaporating and crystallizing. Sulpho-carbolate of zinc is found readily in the drug stores, and consists of colorless, transparent and efflorescent crystals, which are soluble in about twice their weight of water. From the ingredients of this material is it readily observed that it offers a compound that should prove valuable in the treatment of pyorrhea alveolaris in nearly all of its phases. The U. S. Dispensatory says that sulphocarbolate of zinc is an antiseptic, astringent, and stimulant to indolent or foul ulcers and in subacute inflammations of the mucous membrane. Practical experience has proven to me that this remedy acts as an astringent, contracting the gums and the tissues about a loosened tooth more rapidly than any other means employed. It is, therefore, useful when the gingivae are swollen, as for instance, after the setting of crowns or the adjustment of bands in orthodontia. It is useful in fistulous tracts, but should not be forced into tissues with no outlet. I have never observed any detrimental effect upon any of the tissues of the mouth. Being refrigerant, sulphocarbolate of zinc may cause some complaint because of its coldness, but warming the solution will modify this difficulty.

In the treatment of pyorrhea, my treatment is usually a ten per cent solution in water. The solution is colored with Tincture of Cudbear. Give the patient a bottle of this to massage the gums between treatments. Inject the solution into the pocket with a good syringe, having a long, slender needle that will reach the bottom of any pocket. The Sub-Q syringe, with a gold needle, is very good. Carry the point as deeply as possible and flood the pocket and use force enough to carry the fluid to all parts, then quickly massage the gums. Care should be given not to force the solution too hard into the pockets, as that will cause pain, no matter what might be used. After the injection flood the mouth with warm water from the water syringe. Of course, this treatment follows the removal of deposits from the roots of teeth and necrosed bone, if any. This treatment should begin at the first sitting, even though all deposits are not removed, since it may require a number of sittings to accomplish the surgical treatment. Subsequent sittings enable one to discover the location of remaining deposits by the deep or injected color of the gingivae. From one to a half dozen treatments will stop the flow of pus in many cases. My observation is that there is no return of pyorrhea in the majority of cases. I may have been fortunate in having good cases, but in one test case, which was not seen for a full year after the sulphocarbolate of zinc treatment, there was but one tooth in almost an entire mouthful of affected teeth that became affected again. Another case referred to me by a neurologist, in which the tongue had a peculiar sensitiveness upon its side, was also a severe case of pyorrhea. Within a month the pyorrhea disappeared, as well as the aforesaid disturbance, and neither has recurred. This is a case of over a year's standing, but prophylactic treatment is continued monthly in this case.

The first case in which this remedy was used was a small abscess upon the root of an upper incisor. The swelling was about the size of a pea. One injection of the sulphocarbolate of zinc was given, and the following day the gum was flat, and the condition so near normal that it was indeed surprising.

These cases have been mentioned to show the unusual efficiency of sulphocarbolate of zinc. However, it must be stated that all treatment of pyorrhea alveolaris must be preceded by surgical methods, i. e., removal of deposits upon the teeth, necrosed bone, or resorbed roots, having the apex reduced to a needle-like sharpness removed. We must also remember that all cases are not curable.

In conclusion, let me say that having used this remedy for more than a year, it is found to be giving good results, and is growing in favor among some of my professional friends who have tried it.—W. H. Whitsler in *Summary.*

**On the Separation of Platinum, Gold and Silver from a Mixture of Laboratory Filings.**

The method about to be described about separating platinum, gold and silver from laboratory fillings, is the one followed by a well known Parisian firm of assayers. The quantities of the different chemical reagents and substances given throughout this review are the quantities to be employed in treating a weight of fillings of about 100 gm.

*Dissolution.* Place in a glass globe of one-liter capacity the quantity of filings to be treated and a mixture of 150 gm. nitric acid C. P. and 450 gm. hydrochloric acid. Heat the globe over a dull fire or in a sand bath.

*Evaporation.* When the filings have been dissolved, pour out the contents of the glass globe into a porcelain dish and evaporate the fluid slowly to a syrupy consistence. Now add to the syrupy fluid 100 gm. of hydrochloric acid, and cover the dish with a funnel to prevent any portion of the liquid from splashing out on account of the active reaction which takes place upon the addition of the hydrochloric acid. When the reaction is completed, evaporate the solution as in the previous case, to expel the excess of acid.

*Separation of silver.* Add to the liquid 300 gm. of distilled water. and heat for about one hour in order to complete the precipitation of silver chlorid. Filter and collect the filtrate in a glass of 750 ccm. capacity. The precipitate of silver chlorid which remains in the filter is washed three or four times with boiling water, and this water is added to the filtered solution.

*Separation of platinum.* To the filtered solution 150 gm. of pulverized ammonium chlorid are added, and the contents of the glass are stirred with a glass rod and allowed to decant for at least six hours. The platinum will now be found precipitated in the form of chloro-platinate of ammonia. The platinum precipitate is now collected by filtering, and is washed with 200 gm. of cold water, to which 30 gm. of sal. ammoniac have been added. The platinum precipitate and water are again filtered and the liquid is collected in a glass receptacle of one-liter capacity.

*Separation of gold.* In the latter receptacle 100 gm. of crystals of ferrous sulphate are dissolved, stirring the mixture from time to time with a glass rod, and allowing it to decant for eight to ten hours. The gold is slowly precipitated in the form of a brownish powder. The contents of the glass receptacle are now filtered in order to collect the brown powder, which should be thoroughly washed, as in the case of the silver chlorid.

The separation of the precious metals being now completed, it remains to convert the products thus obtained into metallic form.

After having been completely dried the silver chlorid is placed, together with the filter in which it is held, in a crucible, where it is fused with a mixture composed of three times its weight of sodium carbonate and 4 per cent of its weight of charcoal. After the mass has been fused and cooled the crucible is broken and the silver ingot is removed. The chloro-platinate of ammonia is detached from the filter and the paper is burned in a porcelain dish in order to collect the small particles of platinate that may have remained attached to the paper. Into the same porcelain dish the platinate is placed and calcined, heating the dish slowly and progressively and carrying it eventually to a white heat, at which point it should remain until the evolution of white flames has ceased. The gold is treated by the same process as described in the case of silver, with the exception that instead of the sodium carbonate and charcoal mixture, it is fused with three times its weight of borax and once its weight of saltpeter.—L. Lemerle, L'Odontologie, *Dental Cosmos.*

